# Wonderful Discoveries

**Published:** 1887-07

**Authors:** 


### WONDERFUL DISCOVERIES.

The medical journals for the past ten years have given accounts of wonderful discoveries in surgical science and of their application in practice—the filling of large, deep wounds with sponge, and the organization and assimilation of the latter ; skin-grafting, bone-grafting, and the successful adjustment and regrowth of fingers. Recently two other wonderful discoveries have been reported. One is the organization of rubber within the animal tissues ; the other, the organizing of blood-clots, their formation into new tissue, and the application of them to the surer and better healing of surgical wounds.

As to the first, it appears that Prof. Vanlair, of France, had, in a certain case, inserted a drainage-tube, of ordinary gray vulcanized rubber, one and one-fourth inches in length and one-fifth in diameter, and that this, at the end of seven months, seemed to have undergone partial absorption.

But on examining it with a microscope, it was found that the substance of the rubber had become truly organized ; that the lower end of the tube had become fully assimilated to the surrounding tissue, and had wholly lost its original form ; that the part of the tube next above this had lost its original shapeless appearance and had acquired a complex structure, showing fine connecting tissue fibres with cells of various forms between them, and very numerous capillary blood vessels.

Says the Medical Record : "That india rubber can thus become organized is the more remarkable when we consider that it is a pure vegetable exudation, devoid of all structure, and seemingly more calculated to act as a foreign body and so prevent the union of the wounded surfaces, than to undergo organization to and become thus an integral part of the animal tissues."

The other discovery was by Schede, a German expert. The Boston Medical and Surgical Journal says : "His reported results are almost marvellous ; the blood fills the wound cavity completely, clots and is gradually replaced by permanent tissue formation. By this method resection (amputation) of large joints has healed by primary union, and large portions of the articular ends of bones have been removed without impairment of their articular function. Two hundred and forty-one operations are recorded by Schede, nearly all of which have healed under one dressing by primary union."

These operations included the amputation of forty large joints, with thirty-seven recovering, with no change of dressing and no leakage. The wound having been duly prepared, the blood is let in and left to organize, the whole being covered with protective silk and other dressing.

